# A Typology of Livable Communities and Older Adults’ Health in the U.S.

**DOI:** 10.1093/geroni/igaf122.2721

**Published:** 2025-12-31

**Authors:** Kyeongmo Kim, Denise Burnette, Sol Baik, Seon Kim

**Affiliations:** Virginia Commonwealth University, Richmond, Virginia, United States; Virginia Commonwealth University, Richmond, Virginia, United States; University of Virginia, Charlottesville, Virginia, United States; Sunchon National University, Suncheon, Cholla-namdo, Republic of Korea

## Abstract

Neighborhoods with high-quality built environments and social environments are linked with older adults’ well-being. However, research on the complex interplay of neighborhood types and health outcomes is limited, as is the role of functional limitations. This study aims to: 1) identify neighborhood types based on the World Health Organization’s age-friendly community framework, 2) explore the association of neighborhood type and older adults’ health, and 3) assess whether functional status affects this association. We merged data from the 2017 AARP Age-Friendly Communities Surveys and the AARP Livability Index. Our sample included 3,211 adults aged 65 and older; the majority (59%) were female. Participants identified as non-Hispanic White (81%), Hispanic (8%), Black (6%), and a member of another racial/ethnic group (2%). We conducted latent class analysis to identify neighborhood type. We then explored the relationship between neighborhood type and self-rated health, adjusting for individual sociodemographic and health-related characteristics. We identified a four-class model of neighborhood types: “Connected yet Limited Services,” “Service Integrated,” “Healthy Environment Zones,” and “Supportive Social Engagement.” Older adults in “connected yet limited services” and “service integrated” neighborhoods had worse self-rated health than those in socially supportive neighborhoods, especially among those who reported functional limitations. Our findings indicate that older adults with functional limitations particularly benefit from neighborhoods with robust health support and social engagement opportunities, highlighting the importance of designing inclusive and adaptable age-friendly environments to address diverse and changing needs.

